# Doctoral Education in Nursing in Ibero‐America: An Analysis of Its Evolution and Perspectives for the Future

**DOI:** 10.1111/jan.70422

**Published:** 2025-12-07

**Authors:** José Luís Guedes dos Santos, Javier Isidro Rodríguez‐López, Yalena Ortiz‐Anaya, Sandra Milena Aponte Franco, Dolors Rodríguez‐Martín, Paola Galbany‐Estragués, Olga Maria Pimenta Lopes Ribeiro, Thayza Mirela Oliveira Amaral, Alacoque Lorenzini Erdmann, Allison P. Squires

**Affiliations:** ^1^ Nursing Department Universidade Federal de Santa Catarina Florianópolis Brazil; ^2^ Nursing Graduate Program Universidade Federal de Santa Catarina Florianópolis Brazil; ^3^ Nursing Program Simón Bolívar University Barranquilla Colombia; ^4^ Department of Fundamental and Clinical Nursing, Faculty of Nursing University of Barcelona Barcelona Spain; ^5^ Nursing School of Porto Porto Portugal; ^6^ Rory Meyers College of Nursing New York University New York New York USA

**Keywords:** advanced practice nursing, global health, Latin America, nursing research, postgraduate nursing education

## Abstract

**Aim:**

To provide an overview of doctoral programs in nursing offered in Ibero‐American countries to inform regional collaboration and academic development.

**Design:**

This study was a descriptive, document analysis.

**Methods:**

A systematic mapping was conducted using data obtained from official university and program websites, national postgraduate databases, and academic documents. The variables analysed included country, institution, year of implementation, number of faculty and students, course duration, delivery modality, costs, scholarship availability, internationalisation activities, and research lines.

**Results:**

A total of 94 active nursing doctoral programs were identified. Brazil emerged as the pioneer, launching the first doctoral program in 1982, and remains the regional leader, accounting for 43 programs. Most programs are offered by public institutions (76.6%), delivered primarily in face‐to‐face format (64.1%), and emphasise research (90.4%). There has been a consistent upward trend in the establishment of programs since 2000, with notable expansion between 2011 and 2025. Despite this progress, regional disparities persist, along with a lack of data standardisation and a limited presence of professional doctorates. While 69.1% of programs reported international activities, few offer joint or dual degrees. The most common thematic axis, “Health Care and Nursing,” proved to be broad and non‐specific.

**Conclusion:**

The study reveals the expanding landscape of nursing doctoral education in Ibero‐America, while also exposing persistent challenges regarding access, curricular clarity and regional articulation.

**Implications for the Profession and/or Patient Care:**

Doctoral programs are essential for developing research capacity, academic leadership and evidence‐based care. Strengthening these programs could enhance nursing responses to local health needs and promote scientific progress in care delivery.

**Impact:**

This study provides the first comprehensive mapping of nursing doctoral programs in Ibero‐America, highlighting regional disparities and areas for academic collaboration, with potential impact on policy‐making, curriculum development, and the strengthening of research capacity in nursing education.

**Reporting Method:**

STROBE (Strengthening the Reporting of Observational Studies in Epidemiology).

**Patient or Public Involvement:**

No patient or public contribution.

## 1 Introduction

Doctoral education in nursing plays a pivotal role in consolidating the scientific foundations of the discipline and promoting its professional practice. In contrast to generic research degrees, nursing doctoral programs are distinguished by their emphasis on theoretical development, discipline‐specific research, and the creation and integration of knowledge into the science and practice of care (Parse 2015; Kim et al. 2022).

The advent of the inaugural nursing doctoral program occurred in the United States and it was inextricably linked to the overarching movement to establish nursing as an independent scientific discipline. In 1934, Teachers College at Columbia University initiated a Doctor of Education (EdD) program with a particular focus on health education and nursing, signifying the inaugural doctoral‐level training within this field. Nevertheless, its primary focus was on the preparation of educators rather than on the cultivation of disciplinary knowledge. The establishment of the first PhD program formally recognised as dedicated to nursing, with a clear focus on developing nursing‐specific theory and cultivating academic leadership, did not take place until 1947. At that time, New York University introduced the program. This milestone is widely acknowledged as the foundational moment of nursing doctoral education as a legitimate and autonomous scientific enterprise (Ketefian and Redman 2015; Kim et al. 2022).

In the ensuing decades, the North American model served as a template for doctoral education in nursing across other regions, including the United Kingdom, Canada, Australia, and, eventually, Latin America (Ketefian and Redman 2015; Kim et al. 2022). According to Ketefian and Malvarez (2024), the current global estimate suggests that approximately 657 doctoral nursing programs are currently offered across 39 countries (Ketefian and Malvarez 2024). Nonetheless, substantial variation exists globally in the number, structure, and scope of these programs, as well as in the extent and impact of nursing research (Kim et al. 2022).

In this context, the future of nursing doctoral education has become the subject of growing international debate, particularly considering the expansion of professional doctorates and the need to redefine the role and identity of the PhD within the discipline. As Clarke (2024) posits, conducting quantitative and qualitative analyses of existing programs is imperative to ensure that doctoral education remains firmly anchored in the epistemological foundation of nursing, as opposed to being predominantly influenced by functionalist or biomedical logics.

Despite its growing relevance, international scholarship on nursing doctoral education remains limited, making it difficult to develop a comprehensive understanding of its global progress (Kim et al. 2022). A few studies have previously explored the development of nursing research and postgraduate education, with a particular focus on the Latin American context (Hughes et al. 2022; Medina‐Aedo et al. 2024; Dintrans et al. 2024). In order to expand this perspective, the present study focuses on the Ibero‐American region.

Despite the absence of formal legal or institutional status, the Ibero‐American community encompasses approximately nineteen Latin American nations and three European countries: Spain, Portugal, and the Principality of Andorra. The region under discussion is characterised by its transnational nature, which connects former colonial powers and their now‐independent nations through shared languages, primarily Spanish and Portuguese and cultural traditions. It has a well‐established history of academic and political cooperation. In addition to these historical and cultural ties, Ibero‐America faces specific scientific challenges, such as linguistic and cultural barriers that hinder its full integration into the Anglo‐Saxon academic sphere (Ruiz‐Real et al. 2019; Valderrama‐Zurián et al. 2021).

Analysing nursing doctoral programs in this region is, therefore, not only scientifically relevant but also strategically and socio‐politically significant. Such an analysis allows for an assessment of the degree to which nursing research is consolidated as a disciplinary field across different Ibero‐American countries, and whether doctoral programs are aligned with local health priorities in the face of persistent social, economic, and epidemiological disparities. Moreover, this effort can help strengthen South–South and Ibero‐American academic networks, foster institutional synergies, and support the development of contextually grounded models for nursing researcher training.

## 2 Aim

The aim of this paper was to provide a comprehensive and updated overview of doctoral programs in nursing offered in Ibero‐American countries to inform regional collaboration and academic development. This mapping seeks to contribute to a broader understanding of doctoral education in nursing in the region and to support future initiatives for academic cooperation, curriculum alignment, and scientific integration.

## 3 Methods

### 3.1 Study Design

The present study adopted a descriptive, document‐based research design. Descriptive studies are concerned with the systematic collection and organisation of information, as well as the identification of patterns, with a view to characterising phenomena or contexts. Documentary research involves the analysis of materials that have not yet been subjected to analytical treatment, such as institutional reports, regulations, and documents available online (Bowen 2009). The study adhered to the STROBE (Strengthening the Reporting of Observational Studies in Epidemiology) guidelines for cross‐sectional observational studies, where these were applicable.

### 3.2 Study Setting and Recruitment

The sample consisted of programs identified in countries belonging to the Ibero‐American community, covering Latin America, the Caribbean, and the Iberian Peninsula. Active programs linked to institutions recognised by their respective national higher education systems were included, regardless of their administrative nature (public or private), type of delivery (in‐person, hybrid, or virtual), or educational focus (research or professional practice). Interinstitutional or associated programs were also considered, provided there was public evidence of their existence and operation.

### 3.3 Data Collection

The research was conducted between March and June 2025. Data collection was based on information accessed on the institutional websites of the programs, nursing department and faculty pages, official postgraduate databases (such as the Sucupira Platform in Brazil), academic documents (pedagogical projects, regulations, and curriculum matrices), and evaluation reports. For the purposes of collaborative organisation among researchers from different countries, the information was systematised using a Google spreadsheet.

The following variables were extracted: country, university, start year, number of teachers and students, administrative nature, course duration, modality, operating shift, curriculum structure, course cost, scholarship offerings, internationalisation actions, research lines, and declared thematic axes.

### 3.4 Data Analysis

Data were organised into spreadsheets and analysed using descriptive statistics, including crude numbers (*n*) and frequencies (%). IBM SPSS v.27.0 was used for these analyses. Certain variables, such as the number of students, teachers, and cost per semester, were categorised into ranges. Only information reported by the programs was considered when presenting results, which accounts for the variation in the total number of cases (*n*) across different variables. For internationalisation actions, some programs were counted multiple times if they engaged in more than one type of activity. The criteria for inclusion were the report of activities within the last 3 years, utilising the most current information available. Research lines were classified based on title similarity and the frequency of recurring terms, using content analysis to quantify and categorise textual data (Ahmed et al. 2025).

### 3.5 Ethical Considerations

The study respected the ethical principles of documentary research and used only information in the public domain. As it did not involve sensitive data or direct collection from human beings, it was not necessary to submit it to a Research Ethics Committee.

## 4 Results

**FIGURE 1:**
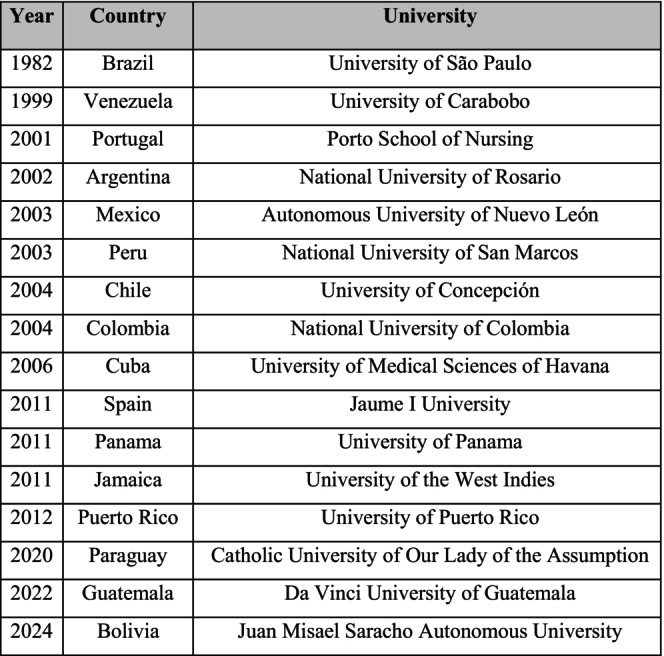
Year of commencement of doctoral programs in nursing in Ibero‐America.

A total of 94 doctoral programs in nursing were identified in Ibero‐America. The chronology of their implementation of the currently active programs shows that the first program was established in Brazil in 1982, followed by gradual expansion in other countries in the region in the following decades (Figure 1).

Table 1 shows the distribution of doctoral programs in nursing according to their regional location. From the 22 countries, 16 have established nursing doctoral programs. There is a higher concentration in South America, particularly in Brazil (45.7%).

**TABLE 1:** Distribution of doctoral programs in nursing by region and country.

Region	Country	*n*	%
Central America and the Caribbean	Mexico	7	7.45
	Puerto Rico	5	5.31
	Guatemala	2	2.13
	Cuba	1	1.06
	Jamaica	1	1.06
	Panama	1	1.06
Ibero‐America	Spain	7	7.45
	Portugal	4	4.26
South American	Brazil	43	45.74
	Peru	12	12.76
	Chile	3	3.19
	Colombia	3	3.19
	Argentina	2	2.13
	Bolivia	1	1.06
	Paraguay	1	1.06
	Venezuela	1	1.06

Regarding the main institutional and curricular characteristics of doctoral programs in nursing, most began after 2000, evidencing the recent growth of doctoral education in the field. Public programs predominate (78.7%) and focus on research (90.4%). In terms of duration, most are expected to last 4 years (59.6%) and are predominantly classroom‐based (64.1%). Regarding the faculty members, the majority of programs have between 11 and 30 faculty, and the number of students per program varies widely, with those with more than 50 students standing out. Most programs operate in the morning and afternoon shifts and follow a semester‐based curriculum (Table 2).

**TABLE 2:** General characteristics of doctoral programs in nursing.

Variable	Category	*n*	%
Year of creation (*n* = 89)	1980–1990	4	4.49
	1991–2000	7	7.86
	2001–2010	26	29.21
	2011–2020	35	39.32
	2021–2025	17	19.10
Administrative nature (*n* = 94)	Public	74	78.72
	Private	20	21.27
Focus (*n* = 94)	Research	85	90.42
	Professional Practice	9	9.57
Course duration (*n* = 89)	4 years	53	59.6
	3 years	26	29.2
	5–7 years	7	7.87
	2 years	3	3.37
Modality (*n* = 78)	In‐person	50	64.1
	Hybrid	22	28.20
	Virtual	6	7.69
Faculty range (*n* = 64)	1 to 10	4	6.2
	11 to 20	25	39
	21 to 30	24	37
	31 to 40	4	6.25
	Over 40	7	10.9
Student range (*n* = 60)	1 to 10	12	2
	11 to 20	12	16
	21 to 30	4	6
	31 to 40	7	11
	41 to 50	6	10
	Over 50	21	35
Working hours (*n* = 41)	Morning and Afternoon	27	65
	Morning, Afternoon, and Evening	7	17
	Morning	4	9.7
	Afternoon	3	7.32
Course offerings (*n* = 75)	Semester	43	57.33
	Annual	20	26.67
	Modular/intensive block	12	16

**FIGURE 2:**
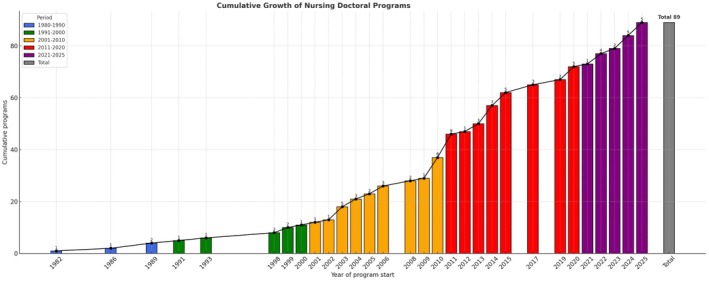
Cumulative growth of doctoral programs in nursing in Ibero‐America.

Figure 2 illustrates the cumulative growth of doctoral programs in nursing in Ibero‐America, highlighting the intensification of the creation of these courses since the 2000s, with an even more significant acceleration between 2011 and 2025.

Table 3 presents information related to program funding, scholarship opportunities, and internationalisation initiatives. About half of the programs are paid (53.2%), with semester costs ranging from USD 500 to USD 4000. Regarding scholarships, 53.7% depend on external funding, while 46.3% offer scholarships directly. Internationalisation efforts are evident in 69.1% of the programs, mainly through academic exchanges and visiting scholars.

**TABLE 3:** Funding, scholarships, and internationalisation.

Variable	Category	*n*	%
Course cost (*n* = 94)	Paid	50	53.19
	Free	44	46.81
Cost per semester (USD) (*n* = 21)	Up to 500 USD	6	2
	501 to 1000 USD	1	4
	1001 to 2000 USD	9	4
	2001 to 4000 USD	5	2
Possibility of scholarship (*n* = 67)	According to external funding	36	53
	Direct offer	31	46
Internationalisation Collaboration (*n* = 94)	Yes	65	69.1
	No apparent	29	30
Internationalisation actions (*n* = 87)	Academic exchanges	42	48.2
	Hosting international visiting professors	28	32
	Offering of international doctoral programs	9	10
	International research networks/projects participation	8	9.2

Of the 94 programs analysed, the main focus or research lines could be identified in 80. Table 4 presents a synthesis of the thematic areas identified across nursing doctoral programs in Ibero‐American countries, highlighting their frequency, core topics, and key emphases. The analysis of these lines of research reveals a significant thematic concentration around the axis ‘Health Care and Nursing’ and ‘Health Management and Policies’.

**TABLE 4:** Classification of thematic research areas, frequency, core topics, and emphases.

Thematic area	Occurrences	Core topics	Emphasis
Health Care and Nursing	77	Care, health, promotion, nursing, caring	Comprehensive care practices at different levels of care, health promotion, fundamentals of care, and clinical and community approaches
Health Management and Policies	35	Management, policies, process, organisation of services, administration	Health governance, planning and evaluation of public policies, management of nursing services and public health
Education and Professional Training	19	Education, training, teaching, teaching‐learning process, research	Professional training in nursing, pedagogical practices, teacher development, educational innovation, undergraduate and graduate teaching
Public Health and Epidemiology	18	Public health, social determinants, epidemiology, prevention, public policy	Health promotion at the population level, analysis of social determinants, primary care and health planning
Clinical and Specialised Health	17	Clinical, chronic conditions, pain, cancer, diabetes, hospital, delirium, rehabilitation	Specialised and technological care, hospital care, clinical practices, management of chronic and acute diseases, and urgent and emergency contexts
Sociocultural and Human Dimensions	3	Addictions, biopsychosocial, surroundings, spirituality, ethics, human rights, subjectivity	Humanised, ethical, and cultural approach to care, attention to diversity and the psychosocial needs of health subjects and collectives

**FIGURE 3:**
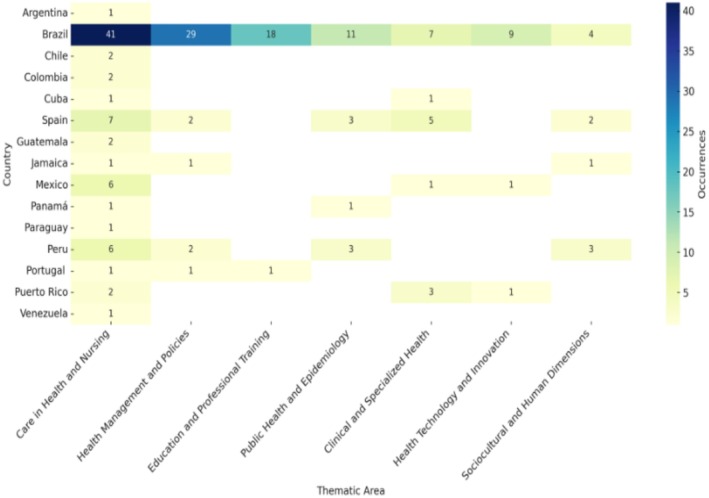
Comparative matrix of thematic areas in nursing doctoral programs by country.

Figure 3 presents a comparative matrix of the thematic areas of doctoral nursing programs across Ibero‐American countries. The thematic areas are sorted in descending order based on the total number of occurrences. Only areas with actual presence are displayed, which enhances the visibility of regional priorities and thematic concentration. The comparative matrix reveals variations between countries. Brazil, for example, has a wide and diversified distribution of axes, with a strong presence in practically all thematic areas. On the other hand, countries with fewer programs tend to focus their lines on general care or priority areas of collective health.

## 5 Discussion

The results simultaneously highlight the expansion and regional inequality in the provision of doctoral programs in nursing in Ibero‐America. Brazil stands out as a pioneer, having started its doctoral training in 1982 and has the largest number of programs today. There has been progressive advancement in the creation of these courses over the decades, with significant growth since the 2000s, reflecting the strengthening of research and advanced nursing training in different national contexts.

This movement is strongly associated with scientific development, public policies to support postgraduate studies, and the consolidation of nursing as an academic discipline. As argued by Kim et al. (2022), the strengthening of doctoral training depends on sustained institutional and national policies, as well as on the capacity for regional integration. In Brazil, for example, the role of CAPES, which is a specific governmental agency for support and evaluation of graduate education, has been fundamental for the institutionalisation of *stricto sensu* postgraduate studies (Rodrigues et al. 2008). Similarly, in other countries in the region, there are initiatives aimed at teacher training, scientific production, and expanding training capacity (Ketefian et al. 2001; Mendoza Otero et al. 2021).

The uneven distribution among countries reflects structural and historical disparities. While countries with a consolidated tradition in higher education and research, such as Brazil, Portugal, Mexico, and Peru, have a greater supply, others still face challenges in implementing or sustaining doctoral programs. Even so, over the last three decades, there has been sustained growth in the creation of these programs, especially within public and private institutions seeking to respond to economic and social demands based on models inspired by US education (Ferriani et al. 2005; Cassiani et al. 2024).

It is worth noting that, although many low‐ and middle‐income countries may lack domestic doctoral programs, several have sponsored nurses to pursue PhD studies abroad under return‐to‐service agreements, requiring them to return to teach and build local research capacity. Such strategies are essential for developing a cadre of nurse scholars capable of generating contextually relevant knowledge and advancing the discipline within their countries. Scholars in global health and nursing have long emphasised the importance of strengthening research capacity among health professionals to promote evidence‐informed practice and sustainable health systems (Edwards et al. 2009; PAHO 2024).

For the purposes of this analysis, only active programs were considered. It is important to note the case of Argentina, where a doctoral program was initially established in 2001 (PAHO 2017) but is no longer active. In addition, as a strategy to expand doctoral education in nursing, several public universities in Argentina joined forces in 2024 to establish a new national interuniversity doctoral program.

Although this study focuses on doctoral programs explicitly designated in nursing, it is important to recognise that many universities also offer interdisciplinary doctoral programs—such as those in Health Sciences or Public Health—that include research lines directly relevant to nursing. While these programs may not fall under the disciplinary label of ‘nursing’, they nonetheless contribute substantially to the scientific development and knowledge production of the field of nursing, as observed in several European countries, where nurses often pursue doctoral studies in related disciplines such as health sciences or public health (López‐Montesinos and Maciá‐Soler 2015; Dobrowolska et al. 2021).

The predominance of public programs with a research‐oriented focus underscores the central role of state universities in scientific training and knowledge development. The classification of program focus was based on the information explicitly provided on institutional websites. Only nine programs were identified as *professional doctorates*, located in Brazil, Chile, and Puerto Rico. However, the Puerto Rican programs operate in partnership with U.S. universities, reflecting the country's political and academic ties as a U.S. territory. Therefore, the professional doctorate modality is currently limited to Brazil and Chile within the Ibero‐American context. All other programs were classified as *research‐oriented*, and it was not possible to analyse hybrid or mixed focuses due to insufficient information.

The limited presence of programs specifically dedicated to advanced practice or innovation in health services reveals a persistent gap between academia and the contemporary challenges of the nursing profession. This gap may hinder the advancement of evidence‐based practice and the integration of care science into solutions for complex problems within health systems. Nevertheless, it is important to acknowledge that academic doctoral programs also contribute significantly to professional practice by developing critical, reflective, and research‐based competencies that can transform care and management contexts. Although professional doctorates have gained international recognition and widespread adoption in Anglo‐Saxon countries since the 1990s (Hughes et al. 2022), their presence in Ibero‐American countries remains incipient. This scenario highlights the need for regional policies that promote the diversification of doctoral education models and strengthen the connection between advanced academic training and the practical demands of health systems.

The curriculum structure remains traditional, with a predominance of face‐to‐face courses and a semester‐based organisation, which contrasts with global movements toward flexibility, modularity, and hybrid teaching. Flexibility in higher education is a multifaceted concept that encompasses time, place, content, pace, and pedagogy, with learner autonomy situated at its core—particularly after the COVID‐19 pandemic, which has led to an unprecedented expansion of digital and hybrid learning models (Barua and Lockee 2024).

The diversity of training models also reveals the absence of shared parameters across the region. Moreover, the concentration of faculty within the mid‐range (11–30 members) and the heterogeneity in the number of students per program reflect both the existing institutional capacity and strategies for program expansion. These aspects warrant further analysis in light of quality assurance principles, student supervision, and academic sustainability.

In terms of cost, although a significant number of doctoral programs are tuition‐free, approximately half require payment, with substantial variation in semester fees. This scenario highlights persistent inequalities in access to advanced education, particularly when the availability of scholarships depends primarily on external funding agencies. Consequently, ensuring equity in both admission and retention remains a structural challenge in many contexts. In countries where constitutions guarantee free public education in official institutions, such as Brazil, this principle has been a key driver in expanding access to higher education. Ensuring free and high‐quality education for all citizens therefore constitutes a *sine qua non* condition for the establishment and preservation of a democratic state (Jucá and Mattos 2021).

The presence of international activities in two‐thirds of the programs demonstrates significant progress toward global engagement. These initiatives include academic exchanges, participation in international research networks, hosting visiting professors, and, to a lesser extent, offering international doctoral programs. Many of these efforts are associated with organisations such as the International Network for Doctoral Education in Nursing (INDEN), the Latin American Association of Schools and Colleges of Nursing (ALADEFE), and other institutional or thematic collaborations (Ketefian and Malvarez 2024).

However, 29 programs showed no evidence of internationalisation, representing both a gap and a strategic opportunity. Strengthening collaboration networks, establishing joint degrees, and developing international research projects could enhance the global visibility of doctoral programs, foster student and faculty mobility, and boost regional scientific production. International competitiveness, recruitment of foreign students, and academic accreditation are key elements for the sustainability of doctoral education, as highlighted by Valenzuela‐Suazo and Sanhueza‐Alvarado (2015) and by UNESCO guidelines (Mendoza Otero et al. 2021). In this sense, fostering synergy between regional networks and global initiatives becomes essential to advance sustainable internationalisation and promote greater equity and reciprocity in doctoral education across Ibero‐America.

The analysis of the research lines of doctoral programs in nursing in Ibero‐America reveals a marked thematic concentration around the axis of ‘Health Care and Nursing’. This predominance reflects the foundational principles of the nursing discipline, reaffirming care as both the object and the method of its practice and research. This emphasis aligns with the historical commitment of nursing to direct healthcare toward individuals, families, and communities. In this context, the challenge for contemporary nursing lies in reestablishing a more conscious and reflective culture of care, one that critically examines everyday actions and responds to the complexities arising from current paradigm shifts. While the focus on care remains essential, there is a growing need to broaden research agendas to encompass more diverse and systemic perspectives on health, policy, and society (Calderón‐Macías et al. 2025).

The second most frequent axis, “Health Management and Policy,” indicates a growing effort to train nurse researchers capable of contributing to service organisation, public policy formulation and evaluation, and the development of innovative management models. This trend may reflect the increasing leadership role of nurses in the governance of health systems, particularly within contexts that emphasise efficiency, quality, and equity. The competencies developed in this domain are also essential for professional and patient advocacy.

Subsequent thematic domains –“Education and Professional Training” and “Public Health and Epidemiology” – underscore the central role of educational qualifications and the understanding of social determinants of health in nursing doctoral education. The emphasis on education highlights initiatives to strengthen pedagogical preparation and educational research, while the public health focus reflects the discipline's enduring commitment to addressing population health challenges through scientific inquiry and community engagement.

The axis “Clinical Health and Specialties” stands out in programs oriented toward advanced practice, care technologies, rehabilitation, and the management of chronic and acute conditions. This represents an expanding area of growth for nursing, particularly in light of demographic and epidemiological transitions across the region. Although less frequent, the “Technology and Innovation in Health” axis signals the gradual incorporation of digital health agendas and technological innovation into nursing research. Finally, the “Sociocultural and Human Dimensions” axis, while less common, encompasses emerging themes such as spirituality, ethics, cultural diversity, and the psychosocial dimensions of care, which together enrich the humanistic foundations of the discipline.

## 6 Study Limitations

The present study is subject to limitations inherent to its documentary nature. The data collection process was informed by the information available on the institutional websites of the doctoral programs, which presented challenges related to the updating, completeness, and standardisation of the information. The absence of direct validation with the institutions may have compromised the accuracy of the data, including but not limited to the number of faculty staff, students, curriculum structure, and internationalisation actions.

It was evident that inequality was present in the transparency and organisation of data across the countries that were analysed. This may have had an impact on the consistency and comparability of the results. The breadth of the data collection, encompassing various Ibero‐American countries, necessitated a focus on linguistic and terminological diversity. This was due to the fact that the nomenclatures employed for variables such as research lines, modality, and scholarships exhibited significant variation. These limitations must be considered when interpreting the results, and they serve to reinforce the need for regional efforts to standardise and increase the visibility of academic information.

## 7 Challenges and Future Perspectives

The findings of this study underscore the need to strike a balance between expanding the availability of doctoral programs in nursing and ensuring their alignment with the specific demands of health systems and the contextual specificities of the Ibero‐American region. A key challenge for the field is to strengthen the relevance of doctoral training so that it effectively contributes to advancing evidence‐based practice and addressing structural health challenges across the region.

The absence of standardisation and the paucity of transparency in public data on programs act as a brake on the monitoring and comparative evaluation of doctoral training. In this context, it is imperative to fortify the policies that govern and promote the availability of contemporary, comprehensive, and readily accessible institutional information. The utilisation of such data can facilitate the formulation of regional cooperation strategies, the alignment of curricula, and the coordination of institutional policies, thereby ensuring the consolidation of robust doctoral training that is ethically grounded and committed to addressing the health needs of populations.

Regarding the research lines, the category “Health and Nursing Care” is worthy of particular note. Despite its frequent appearance, this category is broad and heterogeneous. This broad categorization makes it difficult to identify specific subtopics or consolidated research trends. A more in‐depth analysis, for example of a bibliometric nature, could reveal with greater precision the research focuses of the programs, their thematic gaps, areas of emergence, and scientific consolidation. Furthermore, it is recommended that research lines aligned with strategic themes for the region be strengthened, including but not limited to indigenous health, gender equity, climate change, and digital technologies in health.

The results presented here can also guide funding agencies in the formulation of thematic calls for proposals and programs to support graduate studies. It is important to note the potential for the enhancement of collaborative networks between South–South and Ibero‐American countries, as well as the establishment of joint supervisory arrangements, faculty and student mobility, and the development of multicenter projects and shared scientific endeavours. Initiatives of this nature are imperative in order to promote the internationalisation of doctoral training in nursing, expand its social and scientific relevance, and ensure its institutional sustainability.

## 8 Conclusion

The present study has mapped and characterised doctoral education in nursing in Ibero‐America, highlighting significant advances in expanding the availability of such programs in recent decades, with countries such as Brazil, Mexico and Peru playing a leading role. Concurrently, significant regional disparities were exposed, both in terms of access and academic consolidation. These disparities are indicative of varying levels of scientific development, public investment, and human resources training policies within the region.

The analysis of the programs indicates a predominance of public, face‐to‐face courses with a focus on research and teaching, structured in semester‐based curricula and linked to internationalisation actions. Nevertheless, challenges persist in relation to the transparency of institutional information, data standardisation, and the paucity of programs focused on professional practice. Moreover, the diversity and generality of research lines make it difficult to identify consolidated scientific trends, which indicates the necessity for further studies.

## Author Contributions

José Luís Guedes dos Santos conceptualised the study, coordinated the research team, and led the manuscript writing. All authors contributed to the study design, data collection, analysis, and critical revision of the manuscript. All authors read and approved the final version for submission.

## Funding

Coordenacao de Aperfeicoamento de Pessoal de Nivel Superior.

## Conflicts of Interest

The authors declare no conflicts of interest.

## Data Availability

The data that support the findings of this study are available from the corresponding author upon reasonable request.
